# Is Melanoma a stem cell tumor? Identification of neurogenic proteins in trans-differentiated cells

**DOI:** 10.1186/1479-5876-3-14

**Published:** 2005-03-22

**Authors:** Suraiya Rasheed, Zisu Mao, Jane MC Chan, Linda S Chan

**Affiliations:** 1 Laboratory of Viral Oncology and Proteomics Research, Department of Pathology, University of Southern California, 1840 N.Soto St. Los Angeles, CA 90032-3626USA; 2 Department of Pediatrics, Keck School of Medicine, University of Southern California, 1840 N. Soto St. Los Angeles, CA 90032-3626, USA

## Abstract

**Background:**

Although several genes and proteins have been implicated in the development of melanomas, the molecular mechanisms involved in the development of these tumors are not well understood. To gain a better understanding of the relationship between the cell growth, tumorigenesis and differentiation, we have studied a highly malignant cat melanoma cell line that trans-differentiates into neuronal cells after exposure to a feline endogenous retrovirus RD114.

**Methods:**

To define the repertoire of proteins responsible for the phenotypic differences between melanoma and its counterpart trans-differentiated neuronal cells we have applied proteomics technology and compared protein profiles of the two cell types and identified differentially expressed proteins by 2D-gel electrophoresis, image analyses and mass spectrometry.

**Results:**

The melanoma and trans-differentiated neuronal cells could be distinguished by the presence of distinct sets of proteins in each. Although approximately 60–70% of the expressed proteins were shared between the two cell types, twelve proteins were induced *de novo *after infection of melanoma cells with RD114 virus *in vitro*. Expression of these proteins in trans-differentiated cells was significantly associated with concomitant down regulation of growth promoting proteins and up-regulation of neurogenic proteins (p = < 0.001). Based on their physiologic properties, >95% proteins expressed in trans-differentiated cells could be associated with the development, differentiation and regulation of nervous system cells.

**Conclusion:**

Our results indicate that the cat melanoma cells have the ability to differentiate into distinct neuronal cell types and they express proteins that are essential for self-renewal. Since melanocytes arise from the neural crest of the embryo, we conclude that this melanoma arose from embryonic precursor stem cells. This model system provides a unique opportunity to identify domains of interactions between the expressed proteins that halt the tumorigenic potential of melanoma cells and drive them toward neurogenerative pathways involved in early neurogenesis. A better understanding of these proteins in a well-coordinated signaling network would also help in developing novel approaches for suppression of highly malignant tumors that arise from stem-like embryonic cells.

## Background

Melanomas are a heterogeneous group of tumors that arise in the skin, eye, meninges and other parts of the body. While early stages of cutaneous melanomas are recognized by changes in the size, shape or color of black nevi, most cancer cells grow downward from the skin and invade neighboring tissues before they are detected as highly metastatic tumors in lymph nodes or other organs [1]. Patients with malignant melanomas do not respond well to the currently available therapies and most treatments remain ineffective.

**Figure 1:**
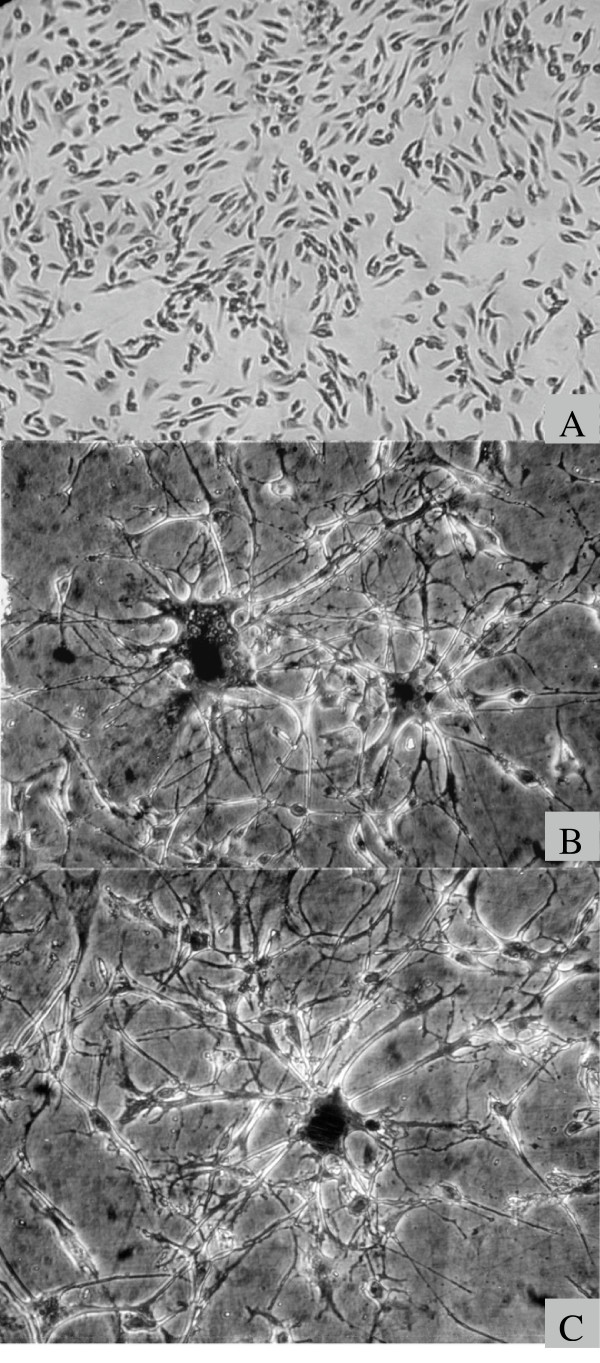
**Photographs of the cat melanoma cell cultures before and after trans-differentiation. A: **Uninfected control melanoma cells 48 hours after culture. **B & C**: Two different cultures of trans-differentiated neuronal cells, 48–72 hours after infection with the endogenous feline retrovirus RD114.

To gain a better understanding of mechanisms involved in the growth, differentiation and transformation of cells, we have studied a fully differentiated (pigmented) highly malignant cat melanoma cell line CT1413, that trans-differentiates into neuronal cells, 48–72 hours after infection with the endogenous cat retrovirus RD114 *in vitro *[2,3]. The trans-differentiated cells exhibit long multipolar cytoplasmic extensions that form a network of connections with giant multinucleated neuronal cells and smaller glial-like cells (Fig. 1A, B &1C). This transformation is a highly specific event for CT1413 cells and its interaction with RD114 virus since no morphological change occurs when these cells are treated with various chemicals known to induce cell differentiation (dexamethasone, insulin, isobutyl-methyl-xathine) or they are infected separately with primate retroviruses that use the same neutral amino acid (NAA) transporter proteins as receptors as does RD114 virus [4,3]. In addition, human, mouse and hamster melanoma cell lines infected with RD114 virus, or CT1413 cells infected with the feline or murine leukemia viruses also fail to exhibit any change in cellular morphology.

Since RD114 virus does not replicate in the terminally trans-differentiated neuronal cells, we hypothesized that binding of virus to its receptor on the melanoma cell membrane could induce conformational changes that generate a cascade of molecular interactions through distinct signals which arrest the tumor cell growth and drive them toward neurogenetic pathways. To test this hypothesis we have analyzed comprehensive protein profiles of both the malignant melanoma and its counterpart trans-differentiated neuronal cells by 2-dimensional gel electrophoresis (2DGE) and identified differentially expressed proteins in the two cell types by Matrix-Assisted-Laser-Desorption-Ionization-Time-Of-Flight mass spectrometry (MALDI-TOF-MS). Herein we show that the trans-differentiation of melanoma into neuronal cells is directly associated with *de novo *expression of pro-inflammatory cytokines, neuro-regulatory enzymes/kinases, neurotrophic factors and concomitant suppression of growth-promoting proteins. This repertoire of proteins is not only responsible for the generation of neuronal cells but it is also involved in the reversion of a highly malignant tumor into non-cancerous neuronal cells.

## Results and Discussion

**Figure 2:**
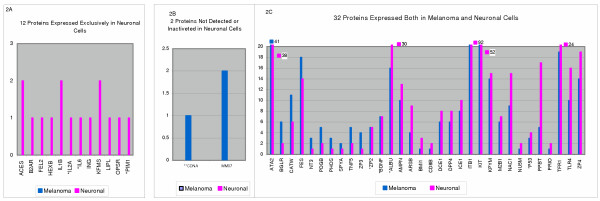
46 proteins identified by MALDI-TOF-MS from multiple gels derived both from melanoma and neuronal cells. **X-axis **= Protein names (abbreviations): **Y-axis **= frequency or # of times a protein is detected and confirmed by mass spectrometry from multiple gels. **Pink **bars represent proteins in neuronal cells and **Blue **bars indicate proteins present in melanoma cells. Protein names, abbreviations and accession numbers are from SWISS-PROT database used for protein identification (See Table 1-3 for details). One asterisk (*) indicates that the protein is detected in single, non-complexed form only in neuronal cells and two asterisks (**) indicate that this protein is detected as single protein only in melanoma cells. **A**: From left to right, expression of 12 proteins detected exclusively in trans-differentiated neuronal cells; 3 proteins were detected in single (non-complexed) form only and are marked by (*) and 9 proteins were expressed as complexes with other proteins. **B**: Two proteins were detected in melanoma cells only (i.e. NOT detected in neuronal cells); CDNA was expressed as a single protein (**) and MMO7 was present in a complex form. **C: **Expression frequencies of 32 proteins expressed both in melanoma and neuronal cells. Left side of the graph displays proteins that are detected at higher frequency in melanoma and the right side shows proteins that are detected at a higher frequency in trans-differentiated neuronal cells.

We have compared 3129 protein spots in 15 gels derived from RD114-infected and uninfected melanoma cells from two independent experiments and analyzed peptide fingerprints of 467 differentially expressed (up-regulated and downregulated) protein spots by MALDI-TOF-MS. A total of 46 proteins were confirmed unambiguously from 302 spots excised from multiple gels of both experiments (Figure 2). The remaining 165 spots did not identify any protein from the SWISS-PROT database or proteins were not identified reproducibly from corresponding spots in different gels. Since species-specific isomers and protein-protein interactions in cat cells may be functionally different from those of human or other species, all 46 proteins reported here were confirmed from the feline protein database.

**Table 1:** Proteins Expressed Exclusively in Neuronal Cells

**Protein Name**	**Abbreviation**	**Accession #**	**Functional Category**
Acetylcholinesterase precursor	ACES	O62763	Membrane Enzyme
Beta-2 adrenergic receptor	B2AR	Q9TST5	Membrane Receptor
Beta-hexosaminidase beta chain precursor	HEXB	P49614	Lysosomal Enzyme
Interleukin-1 receptor beta precursor	IL1B	P41687	Cytokine Receptor
Interleukin-2 receptor alpha chain precursor	IL2A*	P41690	Cytokine Receptor
Interleukin-6 precursor	IL6*	P41683	Cytokine
Interferon gamma precursor	ING	P46402	Cytokine
Macrophage colony stimulating factor I receptor precursor	KFMS	P13369	Membrane tyrosine kinase Receptor
Major allergen I polypeptide chain 2 precursor	FEL2	P30440	Cytoplasmic Cat Allergen
Lipoprotein lipase precursor	LIPL	P55031	Membrane Enzyme
Red-sensitve opsin	OPSR	O18913	Sensory Retinal protein
Proto-oncogene serine/threonine-protein kinase pim-1	PIM1*	Q95LJ0	Serine Threonine Kinase
**B:**			
**Proteins Expressed Exclusively in Melanoma cells**			
**Protein Name**	**Abbreviation**	**Accession #**	**Functional Category**

Cyclin-dependent kinase inhibitor 1	CDNA**	O19002	Nuclear Cell Cycle Protein
Matrilysin precursor	MMO7	P55032	Membrane Enzyme

**A: **shows 12 proteins that were expressed exclusively in neuronal cells (ACES, B2AB, FEL-2, HEXB, IL1B, IL2A*, IL6*, ING, KFMS, LIPL, CPSR and PIM1). One asterisk (*) indicates that the protein is detected in single, non-complexed form in neuronal cells only. **B: **lists two proteins (CDNA** and MMO7) that were expressed exclusively in melanoma (i.e. not detected in trans-differentiated neuronal cells. Two asterisks (**) indicate that the protein is detected in single, non-complexed form in melanoma cells only.

**Table 2:** Higher Detection Frequency in Neuronal Cells

**Protein Name**	**Abbreviation**	**Accession #**	**Functional Category**
Alkaline Phosphatase	PPBT	Q29486	Membrane Phosphatase
Aminopeptidase N	AMPN	P79171	Membrane Enzyme
Arylsulfatase B precursor	ARSB	P33727	Lysosomal Enzyme
Brain-derived neurotrophic factor precursor	BDNF*	Q9TST3	Neurotrophic Factor
Cellular tumor suppressor p53	P53*	P41685	Nuclear
Dipeptidyl peptidase IV	DPP4	Q9N2I7	Membrane Enzyme
Glutamate decarboxylase, 67 kDa isoform	DCEI	P14748	Cytoplasmic Enzyme
Integrin beta-1 precursor	ITB1	P53713	Membrane Receptor
Interleukin-1 beta convertase precursor	ICE1	Q9MZV6	Cytoplasmic Enzyme
Lysosomal alpha-mannosidase precursor	M2B1	O46432	Lysosomal Enzyme
Major prion protein precursor	PRIO	O18754	Membrane Prion Protein
Mast/stem cell growth factor receptor precursor	KIT	Q28889	Membrane Tyrosine Kinase Receptor
NADH-Ubiquinone oxidoreductase chain 5	NU5M	P48921	Mitochondrial Enzyme
Polycomb complex protein BMI-1	BMI1	Q9TST0	Nuclear Protein
Pyruvate kinase, M1 isozyme	KPYM	P11979	Pyruvate Kinase
Serum albumin precursor***	ALBU*	P49064	Cytoplasmic Albumin Protein
Sodium/calcium exchanger 1 precursor	NAC1	P48767	Membrane Calcium Ion Channel (Plasma Membrane)
T-cell surface glycoprotein CD8 beta chain precursor	CD8B	P79336	Membrane Receptor
Toll-like receptor 4 precursor	TLR4	P58727	Membrane Receptor
Transferrin receptor protein 1	TFR1	Q9MYZ3	Membrane Receptor
Zona pellucida sperm-binding protein 2 precursor	ZP2*	P47984	Membrane Receptor
Zona pellucida sperm-binding protein B precursor	ZP4	P48834	Membrane Receptor

Proteins with higher frequency of detection in complex forms in neuronal cells compared to melanoma. Ten proteins were detected in single as well as in complex forms in both melanoma and neuronal cells. These proteins displayed a higher frequency of detection in neuronal cells than in melanoma. Four proteins Fel d 2 /ALBU*, BDNF*, tumor suppressor protein (P53*) and zona pellucida-2 (ZP2*) were detected both in single (*) and complex forms only in neuronal cells. These proteins were present only in complexes in melanoma cells. The ALBU and P53* proteins also showed increased frequency of detection in the neuronal cells than in melanoma (also see Fig 2C*) and BDNF and ZP2 showed similar detection frequencies in the two cell types.

**Table 3:** Lower Detetion Frequency in Neuronal Cells

**Protein Name**	**Abbreviation**	**Accession #**	**Functional Category**
Beta-glucuronidase precursor	BGLR	O97524	Lysosomal Enzyme
Cathepsin W precursor	CATW	Q9TST1	Lysosomal Enzyme
Neurotrophin-3 precursor	NT3	Q9TST2	Neurotrophic Favtor
Phosducin	PHOS	P41686	Cytoplasmic Phosducin
Platelet-derived growth factor, B chain precursor	PDGB	P12919	Growth Factor
Proto-oncogene tyrosine-protein kinase	FES	P14238	Tyrosine Kinase (Cytoplasmic/Transmembrane)
Sarcoplasmic/endoplasmic reticulum calcium ATPase 2	ATA2	Q00779	Membrane Calcium Ion Channel (Endoplasmic Reticulum)
Serine – pyruvate aminotransferase, mitochondrial precursor	SPYA	P41689	Mitochondrial Enzyme
Tumor necrosis factor ligand superfamily member 5	TNF5	O97605	Cytokine
Zona pellucida sperm-binding protein 3 precursor	ZP3	P48832	Membrane Receptor

Proteins that were detected at lower frequencies in neuronal cells (also see Fig 2 C)

By mass spectrometry each gel spot contained either a single protein per spot in a non-complexed form or as a complex of 2–8 proteins in one spot. The frequency of distribution for the 46 proteins among the 302 spots included 82 single protein spots (32 in melanoma and 50 in neuronal cells) and 220 spots containing protein complexes (103 in melanoma and 117 in neuronal cells.

Among the differentially identified proteins, 12 were expressed *de novo *exclusively in neuronal cells, two were detected only in melanoma cells and 32 of 46 (>69%) proteins were shared between the two cell types (Table 1, 2, 3 and Fig 2). Three of the 12 *de novo *expressed proteins in neuronal cells were detected in single, non-complexed form (designated by * in Table 1 and Fig. 2A) and 9 proteins were detected in complexes with other proteins (Fig. 2A). All newly synthesized or upregulated proteins belonged to families of cytokines, neuro-regulatory enzymes/ kinases, sensory, and other signaling proteins (Tables 1, 2, 3). The two proteins that were expressed only in melanoma but not in any of the gels derived from neuronal cells were cyclin dependent kinase inhibitor 1 (P21-CDNA**) expressed in a non complexed /single form and matrilysin or matrix metalloproteinase-7 (MMO7) (Fig. 2B; Table 1B). These proteins have been shown to be critical for maintaining cell growth and metastasis respectively [5,6]. The binomial probability for the distribution of the 12 newly expressed proteins in the trans-differentiated cells compared to melanoma was statistically significant (Fisher's exact test p = 0.007).

Tables 2 and 3 show proteins that are expressed both in melanoma and neuronal cells. Of the 32 shared proteins in melanoma and trans-differentiated neuronal cells, 10 were detected in single as well as in complex forms, 21 proteins were present in complex forms and one protein, the T-cell surface glycoprotein CD8 beta chain (CD8B) was detected in a single form in both cell types but the complex form of this protein was present only in the neuronal cells (Tables 2 &3 ; Fig. 2C). Each of the 32 proteins derived from both melanoma and neuronal cells have been compared and grouped according to their mean/-normalized quantities (i.e. upregulated or down regulated) or according to their higher or lower frequency of detection (i.e. # of times each protein is detected in multiple gel spots). However, no functional significance has been implied in grouping these proteins (Tables 1, 2,3).

**Figure 3:**
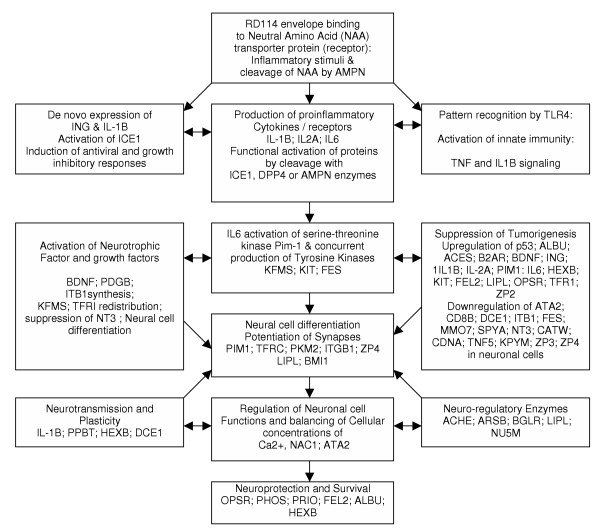
Tentative Signal Transduction Pathways involved in suppression of tumorigenesis and induction of neurogenetic pathways. Proposed protein functions are based on previously published reports.

Analyses of known physiological functions for each of the 46 proteins indicated that 44 of 46 proteins (>95%) expressed in neuronal cells have been shown previously to be associated with the early stages of brain development, differentiation and regulation of nervous system. Based on these biological functions, we have proposed possible roles of these 46 proteins in signaling antiviral responses, arrest in tumor cell growth, modulation of cell membranes and in altering the signal transduction pathways from malignancy to early neurogenesis and cell differentiation (Figure 3).

### Antiviral Responses and Arrest in Tumor Growth

The first line of defense against the RD114 virus infection begins with conformational changes in the melanoma cell membrane due to binding of the viral envelope glycoproteins to its receptor, the NAA transporter protein [4]. The presence of metalloprotease /aminopeptidase (AMPN) on the melanoma cell surface can increase heterodimer formation as it cleaves NAAs from the N-terminus of proteins [7]. The toll-like receptor-4 (Tl-4) recognizes pattern changes in membrane proteins and triggers the innate immunity while the antiviral responses are activated by *de novo *expression of interferon gamma (ING) in the presence of tumor necrosis factor (TNF5) and interleukin-1 beta converting enzyme (ICE1) that were already present in melanoma cells (Fig. 2A&2C). This activates *de novo *synthesis of IL-1 receptor beta (IL-1B), IL-2 receptor alpha chain (IL2A) and IL-6, all of which produce anti-tumorigenic and inflammatory responses. Co-expression of ING, IL-6 and other newly synthesized cytokines prompts proteases (AMPN, ICE1 and dipeptidyl peptidase VI (DPP IV/CD26) to functionally activate these proteins by cleavage [7]. A series of interactions are then initiated by the activated IL-1B and IL-6 toward the suppression of metastatic activity and arrest of melanoma cell growth [8,9]. At this time, two other proteins CDNA/ p21 and MM07 that are essential for tumor growth and metastasis respectively are downregulated and are not detected in trans-differentiated neuronal cells i.e. after the exposure of cat melanoma cells to RD114 virus (Fig. 2B). Suppression of these proteins was associated with concurrent expression of P53 tumor suppressor protein as a distinct single protein in neuronal cells indicating its inhibitory effect on the tumor growth (Fig 2C *). Thus, the antiviral responses of the newly induced cytokines and growth regulatory proteins affect the arrest of tumor cell growth, which then trigger production of neurotrophic factors that alter the pathways responsible for modulation of neurogenesis.

### Activation of kinases, neuro-regulatory enzymes and neurotrophic factors

Both IL-1B and IL-6 are multipotential cytokines capable of activating kinases necessary for phosphorylation of factors involved in the transcription of new genes, synthesis of novel proteins, up-regulation or down-regulation of many existing proteins that enhance signals for tumor suppression and neurogenesis. The cytoplasmic kinase of IL-6 acts synergistically in stimulating production of other kinases and receptors in a ligand-independent manner [10]. In addition, the Pim1 serine/ threonine kinase is expressed as a single non-complexed protein exclusively in neuronal cells (Fig. 2A *; Table 1 group A). This kinase phosphorylates proteins in dendritic and nuclear compartments of stimulated neurons and it is required for long-term potentiation of these cells [11]. The red-sensitive opsin receptor (OPSR) that is normally found in retinal rods is expressed *de novo *in transdifferentiated neuronal cells after exposure of melanoma cells to the RD114 virus (Fig. 2A). This integral membrane protein is phosphorylated on most of its serine and threonine residues present at its C-terminus [12]. It is possible that proteins that are phosphorylated at the serine or threonine residues may also be phosphorylated at tyrosine residues at acidic pH and these may generate distinct neurogenerative pathways [8].

While both melanoma and neuronal cells expressed the mast/stem cell growth factor receptor tyrosine kinase Kit (C-Kit), the frequency of detection for this kinase was significantly higher in neuronal cells compared to melanoma (Fig. 2C). Co-expression of c-Kit with IL-6, IL-2A and IL-B in trans-differentiated cells can further retard tumor growth while promoting cell signaling, differentiation and potentiation of synapses in conjunction with other proteins . The membrane tyrosine kinase receptor (KFMS) for the macrophage colony stimulating factor-1 (CSF-1) was expressed exclusively in neuronal cells and this protein has been shown to protect neural cells from degeneration [13,14]. The FES tyrosine protein kinase was already present in melanoma cells at the time of trans-differentiation and this proto-oncogene has been implicated in morphological differentiation of neuronal cells[15]. The signals produced by IL6 and other cytokines activate brain-derived neurotrophic factor (BDNF*), a protein critical for intracellular protein-protein interactions that result in the development, differentiation, plasticity, and regeneration of neuronal network in the brain [15]. BDNF has recently been shown to be critical for neuronal self-repair following ischemia due to stroke and it contributes to the functional recovery of neurons [16-18].

BDNF binds to tyrosine kinase receptor with a high affinity [19]. It can also auto-phosphorylate and activate other signaling molecules. In our model system BDNF was over-expressed in trans-differentiated cells and it was detected in gel spots both as a single protein and in the form of complexes (Table 2: Figure 2C). Concurrent expression of several kinases, cytokines and growth factors in neuronal cells appears to have enhanced a network of protein-protein interactions between different proteins present in the cell. This is evident by the detection of a large number of detergent-soluble complexes of proteins in these cells (Fig. 2A,B&2C).

**Figure 4:**
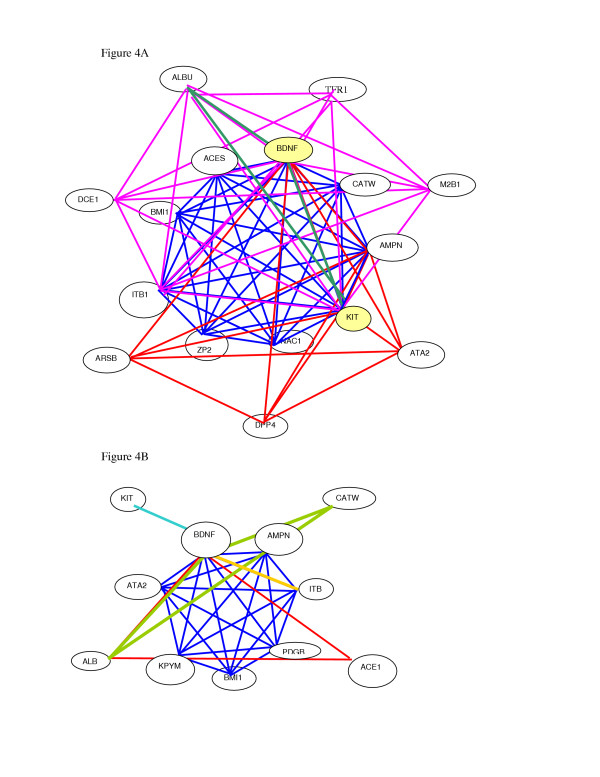
Protein complexes as identified by mass spectrometry in single spots. All proteins identified in one complex are drawn by a single color. **A **represents 4 of the 5 complexes found in trans-differentiated neuronal cells. Each complex contains brain-derived neurotrophic factor (BDNF*) and its interacting partner proteins. Each color in the diagram represents one complex of BDNF with other interacting proteins as identified by mass spectrometry. **NOTE **that all BDNF-interactive proteins in the 4 complexes in neuronal cells contain Kit tyrosine kinase. **B **displays proteins in 5 complexes derived from melanoma cells. **NOTE **that only one complex in melanoma cells contains Kit with no other protein in this complex.

Analyses of all complexed proteins containing BDNF (Fig. 4A&4B) indicated that 4 of 5 complexes (80%) in neuronal cells and only 1 of 7 complexes (14%) in melanoma interacted with tyrosine kinase Kit (p= 0.072 based on 2-sided Fisher's exact test). It is interesting to note that 1 of the 5 BDNF complexes in neuronal cells that did not contain Kit (not shown in Fig. 4), was found to be associated with ZP3, PDGB and TL-4 which acts as an adapter-like protein for BDNF indicating that it was autophosphorylated in the complex without the Kit. This interaction not only stimulates BDNF activity but together these proteins increase length of neurites [20,21]. Although the biological significance of all the recovered complexes from melanoma and neuronal cells is not clear yet, our data suggest that most of the detergent soluble complexes that we have isolated represent functionally active rather than randomly aggregated proteins.

Production of BDNF stimulates expression of acetylcholinesterase (ACES), an enzyme vital for neurite formation and transmission of signals in both the sympathetic and parasympathetic arms of the nervous system [22]. Expression of ACES in glial cells has been associated with the induction of prion (PRIO) protein present in most neural cells [23]. However, since PRIO was already present in melanoma cells and ACES was expressed only after RD114 virus infection, it indicates that ACES does not induce PRIO in these neuronal cells (Fig. 2A&2C). BDNF also activates beta-2 adrenergic receptor (B2AR) in neuronal cells and promotes protein-protein interactions by enhanced binding of different kinases, which trigger astrogliosis and neuronal cell survival [24].

Both beta-hexosaminidase (HEXB) and lipoprotein lipase (LIPL) were expressed *de novo *in trans-differentiated cells (Fig. 2A). These enzymes not only retard cancer cell growth but are also critical for synaptic remodeling in the brain and affect differentiation of neuronal cells respectively [25-27]. The tissue nonspecific isozyme of alkaline phosphatase (PPBT) was expressed with high frequency. This neural cell marker binds to prion protein and it enhances neurotransmission and developmental plasticity [28]. Although PPBT was expressed in both melanoma and neuronal cells its frequency of detection was significantly higher in neuronal cells compared to the tumor cells (Fig. 2 C). Another important enzyme, arylsulfatase (ARSB) that desulfates proteins post-translationally and renders them functional was more frequently expressed in neuronal cells compared to melanoma. This enzyme is important for new glial cells and developing neurons in the brain rather than in mature cells [29].

The pyruvate kinase isoenzyme M1 (KPYM) was downregulated in trans-differentiated cells but its presence is considered important for nerve endings [30]. Likewise the down regulation of neurotrophic factor-3 (NT-3) in neuronal cells is noteworthy since this factor is necessary for the development of mature neurons and enteric nervous system but it is not essential for the generation of newly differentiated neural cells [31]. However, presence of NT-3 has been reported to be essential for enhancement of invasive potential of these tumor cells [17]. Glutamate decarboxylase 67 (DCE1) is a key regulatory enzyme for the neurogenic activity induced by neurotrophic factors [32] and the polycomb complex protein BMI1 is required for stem cell renewal [33] together with Kit, PDGB, NT-3, BDNF and others.

One of the most frequently detected cell membrane proteins, in both melanoma and neuronal cells was integrin VLA-4 beta I subunit (ITB/ CD29). This protein was present in both single and complex forms in both cell types (Fig. 2C). Integrins are cell-surface receptors with multifunctional domains that bind to numerous ligands and transduce signals through a cascade of intracellular events that help in the development of laminae in the central nervous system [34]. In particular, Kit and beta-1 integrins collaborate in modulating cellular functions [35]. During neurogenesis, the synthesis and mobility of integrin is increased by the presence of both BDNF and PDGB [36,37]. These proteins also redistribute transferrin (TFR1) to the membranes of immature neurons, a process essential for neurite formation, axonal cell growth and for regulation of IL-1B production [38,39].

The sarcoplasmic/endoplasmic reticulum calcium ATPase 2 (ATA2) enzyme was detected with a high frequency in both melanoma and neuronal cells and sodium/calcium exchanger 1 (NAC1) a plasma membrane pump was expressed at a slightly higher frequency in neuronal cells than in melanoma (Fig. 2C). Both proteins are essential for neural development, regulation of intracellular concentration of calcium and maintenance of plasticity and synapses, [40].

Expression of different isomers of zona pellucida (ZP2, ZP3 and ZP4) in both melanoma and neuronal cells was totally unanticipated. Although exact function of these sperm-binding receptor proteins in melanoma or neuronal cells is not known, recent reports suggest that ZP domains particularly those of ZP2 that is upregulated in neuronal cells may transduce intracellular signals necessary for polymerization of proteins into neural filaments required for mechano-sensory dendrites [41,42]. It is also possible that ZP proteins may participate in the formation of multipolar extensions in trans-differentiated cells during early neurogenesis. Phosducin (PHOS) is an abundant sensory protein and opsin, is a red-sensitive protein expressed in retinal rods, synapses and photoreceptor cells [43]. Expression of both of these neuro-sensory proteins may be involved in transduction of neurogenic signal in trans-differentiated cells.

## Conclusion

We have shown that RD114 virus infection of cat melanoma cells induces a repertoire of novel proteins that suppress its tumorigenic potential and promote neuronal cell differentiation *in vitro*. To our knowledge, this is the first demonstration that epigenetic stimuli from the cell surface of a naturally occurring melanoma can generate neurogenetic signals that can alter the genome-wide transcriptional and translational programs such that its oncogenic pathways are halted and pro-neuronal auto-regulatory mechanisms are prompted toward neural cell differentiation.

By immunohistochemistry numerous markers of neural cell neoplasms (S-100 protein, neurofilament, epithelial membrane antigen, Leu-7 (CD57), neural specific enolase, neuro-peptide substance P, the low-affinity nerve growth factor receptor (p75NGFR), neural cell adhesion molecule (CD56/N-CAM) and growth-associated phosphoprotein-43) have been shown to enhance malignant potential of melanomas and neuronal cell-derived tumors [44-46]. In addition, peripherin, an intermediate filament protein is expressed during the transition of a neuronal cell to a Schwann cell phenotype and during the normal maturation of epithelioid melanocytes or melanocytic nevi [47]. Based on the immunohistochemical staining, many different tumor types such as neurotropic or desmoplastic melanomas, neuroepithelial and malignant granular cell tumors have also been grouped together as malignant peripheral nerve sheath tumors [48]. None of these proteins was detected in the melanoma or trans-differentiated neuronal cells that we have studied. This could be because we have primarily focused on proteins and profiles (upregulated, down regulated or *de novo *expressed) that distinguish highly malignant melanoma cells from their counterpart trans-differentiated neuronal cells. Our proteomics studies also emphasize that although a significant number of the expressed proteins are shared between the two cell types, distinct protein-protein interactions are operative in altering signals that lead cells toward tumorigenic or neurogenic pathways.

Expression of >69% of germ-line associated sensory or neurogenic signaling proteins, neural-specific enzymes, cytokines, neurotrophic/ growth factors, serine, threonine and tyrosine kinases in cat melanoma cells as well as in trans-differentiated counterpart cells, supports the stem cell origin of this tumor and suggests that the precursor of this melanoma is a stem-like cell rather than the primitive melanoblast committed to melanocytic differentiation only. These cells are unique in that they are fully differentiated (pigmented) and they have the ability to give rise to new cell types that express differentiation-specific proteins. In addition, these cells express many proteins involved in self-renewal of cells. These include the stem cell growth factor receptor protein (C-Kit), PDGB, BDNF, NT-3, polycomb protein 1 and others. Recently, BMI1 has been shown to be essential for efficient self-renewal of neural stem cells [34,49]. As can be seen in Fig 2C, BMI 1 and other protein involved in self-renewal are expressed both in melanoma and trans-differentiated neuronal cells but these are upregulated in neuronal cells. Since both melanocytes and neuronal cells arise from the neural crest of the embryo our studies indicate that CT1413 melanoma is a stem cell tumor.

Based on our findings it can also be speculated that a high incidence of cutaneous melanomas and atypical melanocytic nevi in patients with hereditary neurofibroma, astrocytoma, glioma, meningioma, and other neuronal tumors [50], may also arise due to the expression or suppression of specific embryonic stem cell proteins in these cells. Correlation of cellular phenotypes with signature patterns of proteins in response to epigenetic or environmental changes would now be critical in establishing molecular definitions for classifying clinically related tumors.

To gain a better insight into the complex processes of oncogenesis or neurogenesis, it would also be important to identify proteins in various natural biological settings since most substrate proteins are cleaved by proteases prior to enzyme interactions. Defining the specificity of natural targets may throw light on how biological specificities are achieved in nature. This model system offers a unique opportunity to study protein profiles in relation to specific ligand-binding interactions that are capable of inducing genetic or phenotypic changes in these cells. Characterization of protein-protein interaction domains by the use of constitutively active and dominantly negative constructs and screening of small peptides or interfering RNAs would provide new knowledge into functional significance of each of the proteins that direct cells toward distinct pathways (i.e. oncogenic versus neurogenic). These studies would also have important clinical implications for understanding neurogenerative and neuroprotective mechanisms for neurologic disorders since activated proteins may induce changes in protein subunits in a cell-type specific manner. This information would help in identifying selective inhibitors for tumors and activators/co-activators of neurogenic proteins involved in neural cell development.

## Materials and Methods

### Cell Cultures

A cat melanoma cell line (CT1413) was established *in vitro *from a highly metastatic and melanotic tumor of the lung [2]. Cells were grown in minimal essential medium (MEM) with 10% fetal bovine serum and 2 mM glutamine (growth medium) and were plated in 36 flasks (75 cm^2^), at a density of 2 × 10^6 ^cells/ flask in medium containing 2-ug/ml polybrene [2]. After 24 hrs, culture medium was removed and fresh growth medium containing RD114 virus was added. RD114 is an endogenous retrovirus originally derived from the brain of a young cat and grown in human rhabdomyosarcoma cells [51]. This virus replicates in a wide range of different mammalian cells and does not induce any pathology in healthy cats or in cultures derived from cat embryo cells *in vitro *[52]. The RD114 virus was added to 12 flasks with a multiplicity of infection (MOI) of 1 and another set of 12 cultures was exposed to MOI of 0.25. An additional set of 12 flasks of melanoma cells was cultured without the virus as uninfected controls. After 48 hours post-virus infections, cultures exposed to MOI of 1 showed neuronal cell morphology and this experiment was highly reproducible [3].

To validate protein profiles two independent cell culture experiments were conducted almost 12 months apart and proteomes of both cell types were analyzed separately for differential expression profiles.

### Proteomics Studies

Proteins were extracted from 12 flasks each of the trans-differentiated and counterpart melanoma cells from each of the two experiments. Cells were washed 3× with phosphate buffered saline (PBS) to remove serum and other proteins, and 2 × 10^7 ^cells from each set of experimental and control melanoma cells were removed by the use of a cell-scraper or a "rubber policeman" (i.e. not treated with trypsin to detach cells). Each cell pellet was washed again 2× with PBS and proteins were extracted sequentially by a modified two-step solubilization procedure using two reagents containing different concentrations of 5–8 M Urea, 2%–4% CHAPS, 2 M thiourea and other non-ionic and/zwitterionic detergents (BioRad Sequential extraction kit CA, USA). The most soluble membrane proteins were removed in the first extraction and the less soluble proteins were separated in the second fraction. In order to isolate proteins as close to their natural states in cells as possible, each solubilization step was modified by rapid lysis technique (only 10–15 seconds' cell lysis with 2-seconds' sonication) followed by centrifugation for 90 minutes at 100,000 × g prior to analyzing soluble proteins present in the supernatant by isoelectric focusing (IEF).

All protein fractions from melanoma controls and trans-differentiated neuronal cells were analyzed separately by 2-dimensional gel electrophoresis (2DGE). In the first dimension proteins were separated by IEF on a pH gradient 3–10 and then size fractionated in the second dimension on 6–18% gradient of SDS-polyacrylamide gels. Proteins were stained with Coomassie blue and by the use of a CCD camera and an image-processing analytical program (PDQuest from BioRad) all protein spots were evaluated from each of the 15 gels (9 gels from the first experiment and 6 gels from the second experiments). This program compares both the quality and normalized quantity of each spot across 15 gels and creates a master gel-image of well-calibrated and quantifiable spots using internal reference proteins. A master gel containing 3,129 spots was used to compare each of the corresponding spots in all gels derived from melanoma controls and trans-differentiated neuronal cell. All differentially expressed (i.e. upregulated and down-regulated) proteins were identified in each gel and 467 spots including some spots common to both cell types were excised from multiple gels. Proteins were digested using ultra-pure trypsin and peptide fingerprints of each in-gel digest were analyzed by MALDI-TOF-MS. Full scan mass spectra were acquired and high mass accuracy criteria were used throughout the study with high resolution and strict trypsin specificity. Spectra were submitted to the Protein database (SWISS-PROT) for searching protein identification. In addition to using these stringent criteria, we used manual acquisitions of spectra, which yielded more reliable and reproducible results compared to automated acquisitions using PS1 software. The confidence level in our protein identification was high because almost all proteins were confirmed in corresponding spots in multiple gels and by duplicating the entire experiment for validation. In this study we included only those proteins that were most reproducible in the high stringency Feline Protein database and results were confirmed in other mammalian species (SWISS-PROT). Thus far we have identified 46 proteins among 302 spots from multiple gels tested. All spots in which proteins were not identified reproducibly by mass spectrometry from the same or different gels were NOT included in any analysis.

### Statistical Analyses

The frequency distribution between melanoma and neuronal cells was compared using Chi-square test or 2-sided Fisher's exact test. The mean quantity for each protein was compared between melanoma and neuronal cells using the Mann-Whitney rank-sum test and the probability of a protein occurrence in the trans-differentiated neuronal versus melanoma cells was examined based on the binomial probability distribution with an expected proportion less than 0.05.

## Competing Interests

The author(s) declare that they have no competing interests.
